# Peer support for discharge from inpatient to community mental health services: Study protocol clinical trial (SPIRIT Compliant)

**DOI:** 10.1097/MD.0000000000019192

**Published:** 2020-03-06

**Authors:** Steve Gillard, Stephen Bremner, Rhiannon Foster, Sarah Louise Gibson, Lucy Goldsmith, Andrew Healey, Mike Lucock, Jacqueline Marks, Rosaleen Morshead, Akshay Patel, Stefan Priebe, Julie Repper, Miles Rinaldi, Sarah Roberts, Alan Simpson, Sarah White

**Affiliations:** a Population Health Research Institute, St George's, University of London, Cranmer Terrace, London; b Brighton & Sussex Medical School, University of Brighton, Brighton; c Population Health Research Institute, St George's, University of London; d Health Services & Population Research Department, King's College London, London; e School of Human & Health Sciences, University of Huddersfield, Huddersfield; f Pragmatic Clinical Trials Unit; g Unit for Social and Community Psychiatry, Queen Mary, University of London, London; h Nottinghamshire Healthcare NHS Foundation Trust, Nottingham; i South West London & St George's Mental Health NHS Trust; j Faculty of Nursing, Midwifery & Palliative Care, King's College London, London, UK.

**Keywords:** community mental health services, coproduction, cost effectiveness, discharge, inpatient care, peer support, peer workers, process evaluation, readmission

## Abstract

**Introduction:**

In the period shortly after discharge from inpatient to community mental health care, people are at increased risk of self-harm, suicide, and readmission to hospital. Discharge interventions including peer support have shown potential, and there is some evidence that community-based peer support reduces readmissions. However, systematic reviews of peer support in mental health services indicate poor trial quality and a lack of reporting of how peer support is distinctive from other mental health support. This study is designed to establish the clinical and cost effectiveness of a peer worker intervention to support discharge from inpatient to community mental health care, and to address issues of trial quality and clarity of reporting of peer support interventions.

**Methods:**

This protocol describes an individually randomized controlled superiority trial, hypothesizing that people offered a peer worker discharge intervention in addition to usual follow-up care in the community are less likely to be readmitted in the 12 months post discharge than people receiving usual care alone. A total of 590 people will be recruited shortly before discharge from hospital and randomly allocated to care as usual plus the peer worker intervention or care as usual alone. Manualized peer support provided by trained peer workers begins in hospital and continues for 4 months in the community post discharge. Secondary psychosocial outcomes are assessed at 4 months post discharge, and service use and cost outcomes at 12 months post discharge, alongside a mixed methods process evaluation.

**Discussion:**

Clearly specified procedures for sequencing participant allocation and for blinding assessors to allocation, plus full reporting of outcomes, should reduce risk of bias in trial findings and contribute to improved quality in the peer support evidence base. The involvement of members of the study team with direct experience of peer support, mental distress, and using mental health services, in coproducing the intervention and designing the trial, ensures that we theorize and clearly describe the peer worker intervention, and evaluate how peer support is related to any change in outcome. This is an important methodological contribution to the evidence base.

**Trial registration:**

This study was prospectively registered as ISRCTN 10043328 on November 28, 2016.

## 1 Introduction

It has been well known for sometime that people recently discharged from psychiatric hospital often fail to continue with treatment,^[1]^ relapse, and are readmitted.^[2]^ For example, in the UK it has been reported that 36% of inpatients with psychotic disorders are readmitted within 1 year of discharge,^[3]^ while in New Zealand the readmission rate of all psychiatric inpatients has been reported at 41%.^[4]^ A high proportion of people are readmitted shortly after discharge, with an Australian study of over 35,000 people admitted to psychiatric hospital finding that nearly a fifth of all psychiatric inpatients (half of all people readmitted) were readmitted in the first month post discharge^[5]^ and a UK study of nearly 8000 people observed 15% readmissions within 3 months of discharge.^[6]^ People recently discharged are also at risk of suicide and self-harm with, globally, suicide rates in the first 3 months after psychiatric discharge 100 times the suicide rate in the general population, and suicidal thoughts and behaviours 200 times the level in the general population.^[7]^ In the UK, post-discharge suicides are most frequent in the first week after discharge, with 15% of all suicides nationally among people within 3 months of a psychiatric discharge.^[8]^ Evidence suggests that lack of follow-up care post discharge^[9]^ and lack of continuity of care from hospital to community are predictors of early readmissions,^[10]^ with, conversely, higher levels of follow-up from community mental health services post discharge predicting lower readmission rates.^[11]^

Systematic review evidence suggests that interventions supporting transition from inpatient to community mental health care are feasible and likely to be cost effective.^[9]^ Two of the studies reviewed evaluated multi-disciplinary transitional discharge interventions that included peer support components.^[12,13]^ Peer support in mental health services typically involves the training and employment of people with their own experiences of mental distress and of using mental health services – peer workers – to provide support for others with similar experiences. The studies reviewed above reported reductions in readmissions and use of emergency services compared to care as usual (CAU),^[12,13]^ but it was not possible to separate the contribution of peer support to any effect. An observational pilot of a transitional discharge intervention delivered wholly by peer workers showed promise,^[14]^ while comparison group studies of community-based peer support programs have also reported reductions in readmissions^[15]^ and longer community tenure^[16]^ compared to traditional services alone. A pilot trial of community-based peer mentor intervention for people with a history of recurrent psychiatric hospitalization reported fewer readmissions for people receiving peer mentoring over a 9-month period, compared to community treatment as usual.^[17]^ In another trial, among participants who engaged with treatment, fewer people receiving assertive community treatment (ACT) from a consumer-staffed ACT team reported being hospitalized than those receiving care from a non-consumer ACT team, although length of follow-up varied between participants and was not reported.^[18]^

There is emerging evidence of a wider range of impacts of peer support that might improve psychosocial, as well as service-related, outcomes for people who have had a psychiatric admission. Randomized controlled trials of a peer-supported recovery workbook intervention^[19]^ and a peer-led recovery education program^[20]^ both show improvements in individually reported recovery compared to treatment as usual alone. A recent trial of one-to-one peer support for people experiencing repeated psychiatric hospital admissions reported increased social functioning for the peer support group.^[21]^ There is also increasing trial evidence that one-to-one peer support in mental health services impacts positively on a range of constructs aligned with individual agency, including empowerment,^[22,23]^ self-efficacy,^[24]^ and patient activation.^[25]^ In addition, qualitative research into the mechanisms of peer support indicates the potential role played by peer workers in enabling people to make and maintain wider social networks^[26,27]^ and to act as a bridge between the services users they support and the clinical teams they work alongside.^[28,29]^

While this evidence suggests that interventions employing peer workers might offer a strategy for improving the outcomes of discharge, meta-analyses of trials of a range of peer support interventions have indicated little difference in outcomes for people receiving peer support, in comparison either to treatment as usual or to similar support provided by other mental health workers, once data from across trials are pooled.^[30,31]^ However, those systematic reviews have also pointed to the heterogeneity of the peer support evaluated, issues with the quality of trials, an absence of formal studies of cost effectiveness, and a lack of reporting of how peer support is designed to bring about change in comparison to other forms of mental health support. In particular, inadequate randomization and sequence generation processes,^[30]^ lack of blinding of assessors, risk of bias resulting from missing data, and selective or incomplete reporting of outcomes measured were identified as trial quality issues that need to be addressed.^[31]^

A wide range of qualitative and observation research has indicated that organizational factors such as clarity of job description,^[32,33]^ access to appropriate training and support,^[34]^ shared expectations of the peer worker role,^[33,35]^ and preparation and training for the team that will be working alongside peer workers^[36]^ are all important facilitators of the introduction of the peer worker role. It has been noted how the distinctiveness of peer support, in comparison to other forms of mental health care, can be lost in a formal environment of statutory mental health services if those organizational conditions are not met.^[35,37,38]^ Mental health policy and workforce initiatives in higher income countries are driving the introduction of peer workers into statutory mental health services nonetheless.^[39,40]^ As such, there is a need for high-quality trials of peer support that specify, model, and evaluate the distinctive processes whereby peer support brings about change in specific contexts and settings, as well as for good health economic evaluation. This study aims to address those limitations in the current evidence base while providing clear evidence of the effectiveness and cost effectiveness of a peer worker intervention to enhance discharge from inpatient to community mental health care.

## 2 Methods

### 2.1 Aim

The aim of this study is to establish whether a peer worker intervention to support discharge from inpatient to community psychiatric care reduces psychiatric readmission in the 12 months following discharge, compared to CAU. Participants in the intervention group receive CAU, a Discharge Information Pack, and the peer worker intervention. Participants in the control group receive CAU and the Discharge Information Pack.

We also aim to:

- establish whether the peer worker intervention is cost effective compared to the control condition;
- measure the impact of the peer worker intervention on a range of secondary intra-personal, behavioral, and service use outcomes;
- explore how the processes of intervention delivery relate to outcome.

### 2.2 Study design

This protocol describes a two-group, parallel-group, individually randomized controlled superiority trial, with an allocation ration of 1:1, with trial personnel (outcome assessors and data analysts) blinded to allocation of participants. The protocol described here is protocol version V5.0 dated March 1, 2019.

This trial has been approved by the UK National Research Ethics Service, Research Ethics Committee London, London Bridge, on May 10, 2016, reference number 16/LO/0470, and will be reported in accordance with the Consolidated Standards of Reporting Trials (CONSORT) Statement 2010.^[41]^ Ethical approval for any amendments to protocol will be sought from the Research Ethics Committee and communicated as required by the committee. This trial protocol was created according to SPIRIT guidelines.^[42]^ The trial is registered with the ISRCTN clinical trial register, number ISRCTN 10043328.

Recruitment to the trial began on December 6, 2016, and closed on March 8, 2019. The trial is open to follow-up at the time of submission of this manuscript for publication.

The study was coproduced with people with direct personal experience of mental distress, using mental health services (including psychiatric inpatient services), and of setting up and delivering peer support in statutory and not-for-profit mental health services. For example, site and national “lived experience” advisory panels were involved in developing the intervention handbook and peer support training; researchers with personal experience of mental distress and using mental health services (service user researchers) recruited participants and collected data; members of the research team with personal experiences of mental distress, and of developing and delivering peer support services, were involved in study design.^[43]^ We took this approach to ensure that we addressed issues about the development and evaluation of distinctive peer support identified in the evidence base above.

### 2.3 Internal pilot

An internal pilot trial was conducted in 2 study sites. The pilot aimed to demonstrate that the following conditions could be met:

1. To recruit a total of at least 32 participants (see below) by the end of the pilot period with a recruitment rate of 8 participants per month achieved in both sites for at least 2 months;
2. To demonstrate 90% completeness of electronic patient record data (including primary outcome) of all participants enrolled at end of pilot;
3. To recruit, train, and sustain a team of 2 full-time equivalent peer workers to deliver the intervention at each site.

At the end of the pilot study, a report on progress in achieving the conditions above will be presented to the independent Trial Steering Committee, who will, based on the report, make recommendations for the termination or continuation of the trial.

### 2.4 Participants

Participants in the trial are all inpatients in adult acute psychiatric wards who give written consent to participate in the study. Specific inclusion criteria are as follows:

- Inpatients of adult acute psychiatric wards (acute admission wards and their equivalents as termed locally) with at least 1 previous psychiatric admission in the preceding 2 years;
- Aged 18 years or older;
- Assessed by ward clinical team as likely to be discharged within the next month;
- Capacity to give informed consent to participate in the research.

Exclusion criteria are as follows:

- Having a diagnosis (as recorded in clinical notes) of any organic mental health condition;
- Having a primary diagnosis (as recorded in clinical notes) of eating disorders, learning disability, or drug or alcohol dependency;
- Assessed by ward clinical team as unlikely to be discharged within the next month;
- Assessed by ward clinical team as presenting a current, substantial risk to peer worker.

### 2.5 Setting

The trial is taking place in 7 Mental Health National Health Service (NHS) Trusts – state health service providers – in England. Participants are recruited, and the intervention initiated (for participants allocated to the intervention group), in psychiatric inpatient settings, with data collection and intervention delivery continuing in community mental health services.

### 2.6 Intervention

Participants in the intervention group receive the peer worker intervention, a Discharge Information Pack, and CAU (see below). The peer worker intervention is described in the ENRICH peer support for discharge handbook and accompanying peer worker training manual. Intervention design is underpinned by a change model for peer support in mental health services, developed on the basis of in-depth qualitative research with peer workers, staff who worked alongside them and the service users they supported,^[27]^ and a “peer support principles framework” developed at a preliminary stage of the study to ensure that peer support retains a distinctiveness from other forms of mental health support.^[44]^ Principles focus on:

1. building trusting relationships based on a sense of connection around shared experiences of mental distress and of using mental health services;
2. ensuring that there is reciprocity of listening and learning in the peer support relationship, and that a sense of mutual identity and respect underpins the peer support;
3. validating the experiential knowledge of the participant, including acknowledging the diversity of experiences and understandings of mental health that each person brings;
4. enabling both parties in the relationship to exercise choice and control over how they engage in peer support;
5. enabling social connectivity and realising individual strengths.

As specified in the handbook, potential peer workers are recruited through advert in local mental health services, recovery colleges, not-for-profit mental health organizations, mental health service user and survivor groups, and other relevant community sector organizations in order that the peer worker team at each study site might reflect the communities from which participants are drawn. A job description and person specification specify both the employment experience and personal experience of using mental health services required of the post, as well as the main duties and responsibilities of the role. Potential peer workers are able to attend an information day about the role and project, and have an informal discussion with a member of the study team or site peer worker coordinator (PWC) to assess whether the role is likely to be appropriate for them. The PWC is a key member of the intervention delivery team, has extensive experience working in a peer support or similar role where they have used their personal experiences of mental distress in their work, and has good project and people management skills.

Peer worker training comprises 8 whole-day sessions developed by the study team and a Lived Experience Advisory Panel of people with experience of delivering peer support or of developing and leading peer-led services. The training covers the ENRICH peer support principles and a number of key knowledge and skills sets, identified through literature review and through working with Project Advisory Groups, comprising peer workers and service users, mental health professionals, and members of not-for-profit and peer-led organizations, at each of 6 study sites. Training is led by the site PWC with the support of the study team and by members of staff at the local Mental Health Trust and not-for-profit mental health organizations as appropriate (at least 2 trainers will lead each session). Training adopts a guided, skills-sharing approach with an emphasis on all participants sharing their knowledge and expertise through a range of interactive discussions, role plays, and structured group exercises, with trainees keeping a written reflective log of each session. Training also includes individual feedback sessions with trainers at the mid- and endpoints of the training, group and individual “back to work” sessions with welfare and employment specialists, and at least one inpatient ward visit. At the end of training, peer workers complete induction training with their employer and further ward and community mental health team visits.

In study sites where peer workers are recruited from an existing peer support workforce, peer workers are paid for their time on the training. In other sites, peer workers begin paid employment on completion of the training and on commencing formal employment. All out-of-pocket expenses of attending training are met. Structured “team preparation sessions,” described in the handbook, are held with all inpatient and community clinical teams where participants receive care, facilitated by the PWC, peer workers, and members of the study team. This is to ensure that clinicians and other mental health workers who work alongside peer workers share expectations of the peer worker role.

Trial participants randomized to peer support are assigned a peer worker by the PWC who introduces the participant to the peer worker, as soon as possible after allocation and prior to discharge. Peer workers are matched to the participant by gender where this is specifically requested by the participant or where this is felt to be appropriate by the clinical team; otherwise no additional matching is undertaken. Participants have at least one meeting with the peer worker prior to discharge, and continue to meet weekly until discharge if discharge is delayed (where this delay exceeds a month, contact drops to a brief fortnightly meeting until discharge). Once discharged, participants have a weekly meeting with the peer worker for 10 weeks, stepping down to 3 further fortnightly meetings (i.e., 13 meetings over a 4-month period post discharge). Meetings are flexible in length, as agreed by participant and peer worker. Peer workers have sufficient time in their work plans to spend half a day per contact, including traveling to and from the meeting, although it is expected that meetings typically range from 60 to 90 minutes. Face-to-face meetings can be supplemented by phone calls, using phones provided by the employer, within the peer worker's working hours.

Informed by the domains of the Peer Support Principles Framework,^[44]^ in initial meetings peer workers focus on making a connection with participants and on building the relationship. As part of the training, peer workers are equipped with a range of skills and tools with an individual strengths-based focus, and around mapping and appraising community-based resources and social supports. In subsequent meetings, peer workers and participants agree between them which approaches and tools they will use and are free to identify other tools or develop their own. Once in the community, peer workers do not meet participants in their own homes; the focus is on being alongside the participant as they engage, or re-engage, with a range of social spaces and activities in the community. Peer workers use their experiences of mental distress and of using mental health services as a point of connection with the participant, and to validate and encourage the participant to reflect on their own experiences. Peer workers are encouraged, through training and supervision, to listen to and learn from the participant's experiences as part of a reciprocal relationship, especially with respect to understanding each other's diverse experiences of mental health, in relation to culture, gender, sexuality, and so on, as appropriate. There is an emphasis on the peer worker enabling the participant to access social supports, rather than on the peer worker providing support (as a support worker, for example), in order that dependency in the relationship is minimized, with the ending of the peer support discussed at regular intervals. Peer workers are able to attend discharge meetings, case reviews, and care planning meetings, or to attend appointments with mental health professionals with the participant, at the participant's request.

The handbook specifies the support to be received by peer workers. This focuses on the regular group supervision provided by the PWC – with a focus on using personal experiences of mental distress in the peer worker role – with additional individual supervision as necessary. Peer workers constitute their own team and can be line managed by the PWC, or by another mental health worker (where the PWC lacks sufficient experience to manage a team). The peer worker team is enabled to meet together as agreed to provide each other with peer support. The PWC should receive appropriate supervision and support to fulfil their role.

The Discharge Information Pack provides written information and contact details about Mental Health Trust and community (not-for-profit) sector services locally and nationally that participants might find useful. Participants are given a copy of the pack at allocation and peer workers are able to make use of the pack as they feel helpful.

#### 2.6.1 Measures to improve adherence

To support adherence to the intervention, PWCs at all study sites participate in a regular (6 weekly) Action Learning Set for the duration of the trial, facilitated by a member of the study team, at which they discuss and problem solve issue relating to delivery of the intervention. Issues can be followed up by PWCs through weekly group supervision sessions they lead with peer workers, or by the study team with the Mental Health Trust at each site as appropriate.

### 2.7 Control

Participants in the control group receive CAU and the Discharge Information Pack. CAU post discharge from inpatient psychiatric care is mandated nationally in England as follow-up from community mental health services within 7 days of discharge. Participants in the control group are given a copy of the pack at allocation, with no further instruction specified about how the pack is to be used. Participants in the control group are given the pack in order to control for any effect of information alone as the impact of information is not what is being tested in this study; that is, the aim is to make sure that any effect is due to peer support and not one group having access to more information.

### 2.8 Assessments

#### 2.8.1 Primary outcome

The primary outcome for the trial is psychiatric inpatient readmission (of any type, voluntary or compulsory, as recorded by site) in the 12 months post discharge from the index admission (T2).

#### 2.8.2 Secondary outcomes

Selection of secondary outcomes was guided by the change model for peer support in mental health services previously developed by the research team.^[27]^ Outcomes are collected as indicated below:

- Strength of Social Network as measured using the Social Contacts Assessment,^[45]^ designed to collect data on both the frequency and quality of a range of types of social contacts in the preceding week, at end of intervention, 4 months post discharge from the index admission. (T1)
- Subjective quality of life as measured using the Manchester Short Assessment of Quality of Life,^[46]^ a well-validated patient-reported measure of quality of life, widely used in mental health studies, that has been shown to be sensitive to change. (T0, T1)
- Social inclusion as measured using the Social Outcomes Index (SIX),^[47]^ designed as an objective measure of social inclusion that can be easily extracted from items included in the Manchester Short Assessment of Quality of Life^[46]^ and an additional structured question about friendships. (T0, T1)
- Hope for the future, as measured using the Herth Hope Index,^[48]^ a self-report measure originally developed for use with older adults with chronic conditions and now increasingly used for adult mental health studies. (T0, T1)
- Severity of psychiatric symptoms, as measured using the Brief Psychiatric Rating Scale,^[49]^ a well-validated and widely used measure of clinical severity and the most frequently used severity measure in peer support trials to date. (T0, T1)
- Total number of psychiatric inpatient admissions (any type, voluntary or compulsory, as recorded by site). (T0, T2)
- Time to (first) readmission measured in days post discharge from index admission. (T2)
- Type of psychiatric admission (voluntary or compulsory, as recorded by site) for first readmission. (T2)
- Days in hospital (for all psychiatric admissions). (T0, T2)
- Use of accident and emergency (A&E) for psychiatric emergency measured as number of episodes of liaison psychiatry contact in hospital A&E. (T0, T2)
- Number of contacts with Crisis Resolution & Home Treatment Team. (T0, T2)
- Serious adverse events (SAE), as recorded in the SAE CRF (see below). (T2)

#### 2.8.3 Health economic outcomes

Outcomes are collected as indicated below:

- Quality-adjusted life years, calculated using EQ5D-5L,^[50]^ as widely applied in health economic evaluations of healthcare interventions. (T0, T1)
- Number of contacts and type of professional for community mental health team and mental health crisis services not otherwise listed under secondary outcomes above. (T0, T2)
- Number and type of contacts with non-NHS mental health services, as measured using a modified version of the Adult Service Use Schedule. (T0, T1)^[51]^
- Number, length, and type of contact with peer workers, measured using a structured online questionnaire.
- Number, length, and type of non-participant contact activities (e.g., supervision, training, office-based tasks) undertaken by peer workers and PWCs in delivering the intervention, measured using a structured online questionnaire.

#### 2.8.4 Process measures

Measures of intervention process were informed by the peer support change model referred to above,^[27]^ and were collected as follows:

Strength of therapeutic relationship between participant and a) mental health professional with whom the participant has most contact, measured from the participant's perspective using the STAR Patient version and b) peer worker, measured from the participant's perspective, about the peer worker, using the STAR Patient version and from the peer worker's perspective, about the participant, using the STAR Clinician version.^[52]^ (T1)
Experience of stigma within mental health services, as measured using the stigma subscale of the Barriers to Care Evaluation (BACE-3) Stigma Subscale.^[53]^ (T0, T1)
Internalized stigma, as measured using the Questionnaire on Anticipated Discrimination (QUAD).^[54]^ (T0, T1)
Other peer support received, measured using a set of structured questions. (T0, T1)
Number, length, and type of contact with peer workers, as measured for the health economic evaluation above.
Socio-demographic data are also collected at T0.

### 2.9 Data collection methods

All mental health services use data, including the primary outcome, and secondary and health economic outcomes as listed above are extracted from the site (Mental Health NHS Trust) electronic patient record (EPR) by site information management staff using a pro forma and indicating participants by each site's EPR number for each participant. Data collected at T0 are for the period of 12 months preceding the index admission. Data collected at T2 are for the 12 months post discharge from the index admission.

Online survey data on participant contact and non-contact activities are collected using Limesurvey software. Participant contact activity surveys are completed weekly by all peer workers for the duration of delivery of the intervention. Non-contact activity surveys are completed by peer workers and PWCs for 6 randomly selected weeks during the course of delivery of the intervention. Links to the survey are emailed directly to peer workers and PWCs.

All other data are collected by researchers through face-to-face interviews with participants. At T0, once the participant has been entered into the trial and before randomization, the researcher will, in an appropriate location on the inpatient ward where the participant was recruited, complete the baseline (T0) assessment booklet with the participant comprising measures as listed above.

At T1 (end of intervention) – no sooner than 4 months (120 days) post discharge from the index admission and no later than 6 months (180 days) post discharge from the index admission – the researcher will, in an appropriate location in the community (or on an inpatient unit if the participant has been readmitted at that point), complete the end-of-intervention assessment booklet with the participant, comprising measures as listed above. Participants will be asked, at recruitment, to provide their preferred contact details. Where a participant is not contactable, the participant's clinical team will be asked to contact the participant on the study team's behalf. Researchers will make a maximum of 5 attempts to contact each participant.

**Figure 1:**
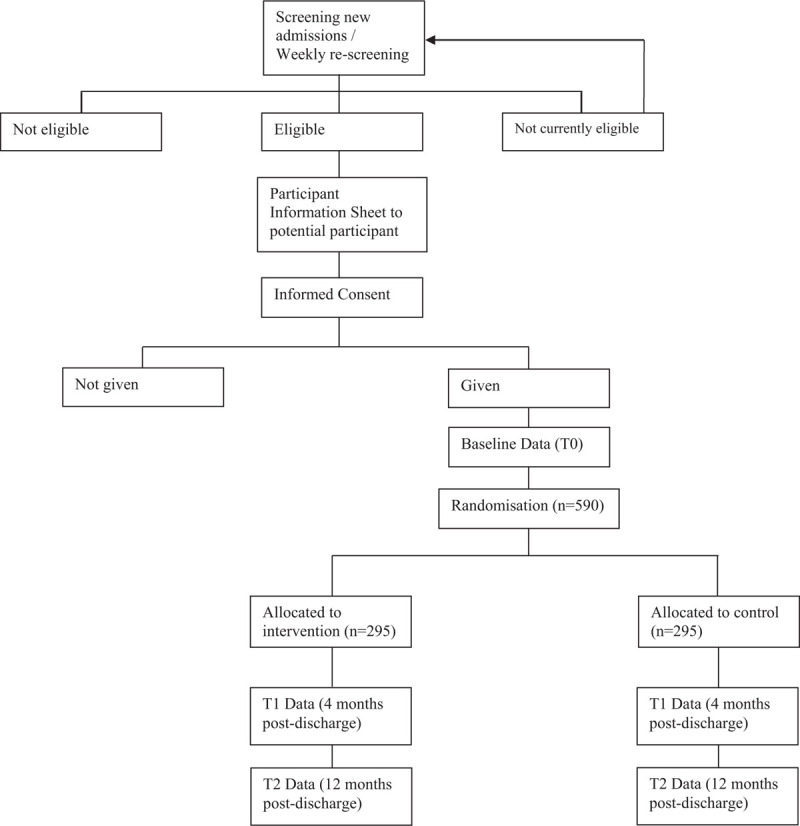
Trial procedures.

Recruitment, randomization, allocation, and assessment procedures are indicated in Figure 1.

### 2.10 Sample size

The sample size has been determined taking into account assumed clustering by peer workers in one group only.^[55]^ No existing data is available on the intracluster correlation coefficient (ICC) in this context so an ICC of 0.05 is assumed. Using data from the first 6 months of 2012 for 3 participating trusts, the average 1-year readmission rate was 34%. Pilot studies of peer worker interventions indicate relative reduction in readmission rates of up to 50%.^[15]^ A systematic review of interventions to support discharge indicates significant absolute reductions of between 13.6% and 37%.^[9]^ To be able to detect a 12% absolute reduction in readmission rates to 22% in the peer worker group, with 80% power at the 5% significance level, 530 patients are required, 270 in the control group and 260 in the peer worker group, assuming an average cluster size of 10 patients and therefore 26 clusters (peer workers).

We considered completeness of primary outcome data at 12 months for people recently discharged from inpatient care in the lead Mental Health Trust, and for a similar population recruited to the FIAT trial.^[56]^ In both cases primary outcome data was available for approximately 90% of the sample. Allowing for a 10% inflation of sample size to account for missing data, we would need to recruit 590 participants (290 in the intervention group and 300 in the control group, assuming an average cluster size of 10 patients and 29 peer worker clusters).

### 2.11 Recruitment

Participants are recruited from adult acute psychiatric wards at Mental Health NHS Trusts. All new admissions to the ward are screened for eligibility on a weekly basis. Where potential participants are identified as currently ineligible (i.e., as currently unlikely to be discharged within a month, or currently posing a substantial risk to the peer worker) they will be re-screened on a weekly basis, in consultation with the ward clinical team, until either deemed eligible or discharged. Existing formal diagnoses using ICD-10 criteria, as recorded in individual patient records, are used for screening purposes.

Eligible potential participants are approached by a member of the clinical team and asked if they are interested in taking part in the study, and if so, given a copy of the study Key Facts sheet and Participant Information Sheet. Where potential participants express further interest in the study, a meeting is arranged with a researcher. At that, or a subsequent meeting, potential participants are invited to give written, informed consent to participate in the study, and having done so complete the baseline (T0) assessments with the researcher.

Following completion of baseline assessments, participant data relating to stratification is passed to a member of the research team not involved in assessments or analysis of data. Randomization is be requested using an online randomization service. Allocation is be communicated to the participant by a member of the research team, in person, not involved in assessments or analysis of data or by the PWC at the site (contacted by telephone from the central study site). Where a participant is allocated to the intervention group, the PWC will make arrangements for a peer worker to meet the participant and begin the intervention, as described above.

### 2.12 Randomization and sequence generation

Participants are to be randomized to the treatment groups in a 1:1 ratio. Randomization is stratified by site and diagnostic group using randomly permuted blocks of randomly varying length to conceal the allocation sequence and achieve the allocation ratio. Site group has 7 strata (the 7 study sites). Diagnostic group has 3 strata: Psychotic Disorders – ICD-10 diagnoses F20–29; Personality Disorders – ICD-10 diagnoses F60; other eligible disorders.

### 2.13 Blinding

All trial personnel are blind to allocation of participants, with the exception of designated team members involved in communicating allocation to participants (as described above). These team members are not involved in assessment or analysis of trial data. As noted above, all collection of secondary and process outcome data by face-to-face interviews at T0 precedes allocation. All trial and site personnel involved in collection of electronic patient record data, including primary outcome data, are blind to allocation of participants. All trial personnel involved in collection of secondary and process outcome data by face-to-face interview at T1 (end of intervention) are blind to allocation of participants. The following measures are designed to ensure blinding of data collection at T1 is as robust as possible:

1. Participants are asked at the beginning of the interview not to reveal their allocation to the researcher;
2. Participants self-complete paper copies of the Manchester Short Assessment of Quality of Life^[46]^ and Herth Hope Index^[48]^ at the beginning of the interview and place these into an envelope unseen by the researcher prior to sealing the envelope. The participant's Study Identification Number is indicated on the paper copies of measures and the outside of the envelope. This is to ensure that a minimum amount of data is collected with the study team blind to allocation should the participant reveal their allocation during the interview;
3. If the researcher becomes unblinded, or suspects that they have become unblinded during the course of the interview, at the end of the interview the researcher completes a Blinding of Interview Form, which indicates:
  a. Whether allocation was revealed by the participant and the number of the question in the interview at which allocation was revealed, or
  b. Whether the researcher suspected that they had become unblinded during the interview and the number of question in the interview at which allocation might have been revealed, and
  c. If so, to which group of the trial the researcher thinks the participant had been allocated.

### 2.14 Data management and monitoring

All data is handled in accordance with the UK Data Protection Act 2018 (incorporating the EU General Data Protection Regulation). Data from face-to-face interviews is entered into Case Report Forms (CRFs) and does not bear the participant's name or other directly identifiable data. The participant's study identification number only is used for identification. A Participant Identification Log, stored securely and separately from the CRF, is used to cross reference participant-identifiable information only as necessary. At the earliest possible opportunity following completion of the interview, the researcher manually enters data from the paper CRF into an online CRF. Data collected from EPR by information management staff at study sites is matched to the participant through their study identification number, and entered into the online CRF by trial personnel. Any transfer of confidential trial data will be by secure upload facility.

All data entered into the trial database via online CRF is subject to appropriate validation (number, date ranges, etc) to ensure that the resulting dataset is of the highest quality, and to minimize any data entry errors. Soft validation will give warning where data is outside of expected ranges and raise queries where such warnings are not heeded. The database has a robust data query management system inbuilt with a full audit trail to track queries to resolution. The database is hosted on secure university-based servers, which comply with robust security standards for clinical data and are subject to daily backups and regular offsite backups. Extracted data will only be made available to the statistical team for analysis on secure servers via a remote desktop. A data management plan will be developed, which will act as a central reference for all data management information relating to the trial.

An independent trial quality assurance manager will conduct a study risk assessment and, based on the risk assessment, produce an appropriate study monitoring and audit plan. Regular audits of trial documentation will ensure that the trial master file is being conducted according to regulatory requirements.

An independent Data Management Committee (DMC) will meet regularly during the trial to review the accruing trial data and assess whether there are any safety issues (see *Trial Safety* below) that should be brought to the attention of the trial team, or any reasons for the trial not to continue, making recommendation to the study team and Trial Steering Committee as necessary. The DMC will be the only body that has access to unblinded trial data, with reports to the DMC prepared by an independent statistician.

### 2.15 Statistical methods

Participant flow through the trial will be shown in a flow chart as per the CONSORT Statement 2010.^[41]^ Baseline characteristics will be summarized for each treatment group by the mean and standard deviation or median and interquartile range for continuous variables as appropriate, and the number and percent for categorical variables.

All analyses will be conducted according to the intention to treat principle, meaning that all randomized participants with a recorded outcome will be included in the analysis, and analyzed according to the treatment to which they were randomized. We will also estimate the complier average causal effect for the intervention on the primary outcome (where compliers are participants who have had at least two peer worker meeting, at least one of which is in the community following discharge). Participants who withdraw consent for their data to be included in the analysis will be excluded from all analysis.

For the analysis of the primary outcome and each secondary outcome, we will present the following information:

- The number of participants in each analysis, by treatment group
- A summary statistic of the outcome (e.g., number (%)), by treatment group
- The estimated treatment effect
- A 95% confidence interval for the estimated treatment effect
- A two-sided *P*-value

For all analyses, a significance level of 5% will be used. Analysis will be conducted using Stata Version 14^[57]^ or higher and R.

The primary outcome (readmission to psychiatric inpatient care in the 12 months post discharge from the index admission) will be analyzed using a mixed-effects logistic regression model with participants clustered by peer worker in the intervention group and participants being their own cluster in the control group.^[55]^ The model will be adjusted for the stratification variables (site and diagnostic group). We will also adjust for pre-specified baseline covariates considered by the trial team to be strongly prognostic of the primary outcome (e.g., ethnicity), and the final set of variables will be fixed prior to un-blinding of treatment allocation.

A full statistical analysis plan, additionally detailing analysis of the secondary outcomes, handling of missing data, and pre-planned subgroup analyses will be agreed and signed off prior to unblinding and final data analysis.

### 2.16 Health economic analyses

The primary economic analysis will take the NHS/Personal Social Services perspective preferred by the National Institute for Health and Clinical Excellence.^[58]^ It will focus on evaluating the net impact of the provision of peer support on resource use over a 12-month period of follow-up, accounting for the cost of contacts with community-based mental health services and any readmission to hospital. A secondary economic analysis will be undertaken using resource utilization and quality-of-life data collected at 4 months post discharge from hospital. This will evaluate the short-term “societal” cost consequences of providing peer support on service use within and outside of the mental health sector (e.g., contact with the police) alongside the extent of any short-term gains in quality-of-life adjusted time spent living in the community (QALYs).

### 2.17 Process evaluation

Using in-depth, qualitative interview data, and process and secondary outcomes data as detailed above, the process evaluation will explore a number of pre-specified change mechanisms that theorize how the intervention may work. The proposed mechanisms are based on previous qualitative research undertaken by the study team,^[27]^ further developed in collaboration with the LEAP group.

Qualitative interviews are conducted with a subsample of 35 (5 per study site) participants in the trial, between end of intervention and 2 months after the end of intervention, with all peer workers (n = 28; approximately 4 per site), 4 and 12 months after they begin working in the peer worker role, and with all PWCs (n = 7; 1 per site), 12 months after peer support was first delivered at the site.

Interview transcripts will be analyzed thematically, combining inductive and deductive approaches^[59]^ in order to explore, experientially, our proposed changes mechanisms, and interpretively, integrating the various clinical, academic, and experiential perspectives of members of the study team into the analytical process.^[60]^ Structural equation modeling will be used to test quantitatively the proposed mechanisms.

### 2.18 Trial safety

Adverse events, of any kind, that might be related to either the trial intervention or trial procedures are logged in an adverse event log. This includes events that are identified, unsolicited, by any trial personnel engaged in delivery of the trial and events that are identified by site clinical studies staff through regular inspection of the EPR of trial participants. Where adverse events are potentially serious, these are entered into a serious adverse event (SAE) CRF. All SAEs are followed up by the site principal investigator (PI) until resolution. At the earliest opportunity, the SAE CRF is forwarded by the PI to the trial manager and chief investigator (CI), and reported to the trial DMC every 6 months. The chair of the DMC makes a formal recommendation in relation to continuation of the trial to the Trial Steering Committee on a 6-monthly basis. SAE includes any adverse event or untoward medical occurrence in a trial participant, whether it is considered to be related to the intervention or not, which results in any of the following:

Death

- Threat to life (places the participant, in the view of the PI, at immediate risk of death)
- Self-harm that is potentially life threatening
- Hospitalization resulting from a medical condition (other than the participant's index condition(s) – as recorded at baseline – unless there are reasonable grounds for concluding that the event was in part or whole caused by the intervention)
- Persistent or significant disability or incapacity (substantial disruption of one's ability to conduct normal life functions)
- Incidents related to the intervention that result in physical injury or other forms of injury or abuse of trial participants or intervention staff (including, for example, sexual contact between participants and intervention staff).

If the SAE is suspected by the site PI to be both related to the intervention and unexpected, notification of the approving NHS Research Ethics Committee (REC) will be expedited, to be received by the REC no later than 15 days after the CI first becoming aware of the SAE. Follow-up reports will be completed within acceptable time frames and sent as detailed above until the reported event is considered resolved.

All deaths (occurring prior to 1 year post discharge follow-up) are reported to the chair of the Trial Steering Committee irrespective of whether the death is related to disease progression, the intervention, or an unrelated event. This report is immediate.

## 3 Discussion

This paper describes, what is to date, the largest individually randomized trial of one-to-one peer support in mental health services internationally, and one of the few trials of peer support which integrates a formal cost-effectiveness study. Peer support is rapidly being introduced into mental health services internationally – in the form of mutual peer support groups^[61]^ as well as the one-to-one peer support we are interested in here – with healthcare workforce policies advocating for the employment and training of a large number of new peer workers.^[39,40]^ Yet as we have seen, the evidence base for peer support remains equivocal,^[30,31]^ with issues relating to both trial quality and a lack of clarity about what peer workers do, how that is distinctive from what other mental health workers do, and how peer support brings about change in outcome. This study attempts to address these issues and hence improve the utility of evidence for policy makers and service providers.

With respect to trial quality, while it remains unfeasible to blind participants to allocation in a peer support trial, we have specified the blinding of assessors,^[31]^ including measures to protect blinding in as far as is practicable, as well as detailing the randomization and sequence generation processes to be employed.^[30]^ We have developed a clear understanding of how peer support, and in particular the relationship between the peer worker and participant, is distinctive from other forms of mental health support and conventional patient-clinician relationships, as articulated in our *principles of peer support* framework,^[44]^ and used this framework to inform the development of the peer worker handbook and training program (the intervention manual). We hope, as a result, that we will be able to clearly report and evaluate – through our process evaluation – how the specific mechanisms of peer support bring about any change in outcome that we might observe. As such, our selection of secondary and process outcome measures is theoretically driven by a change model for peer support in mental health services.^[27]^

In order to ensure that we understand and can adequately report on this distinctiveness of peer support, both in the development and evaluation of our peer worker intervention, we included in our study team and advisory groups a number of people with direct personal experiences of mental distress, using mental health services, and developing and delivering peer support. This “coproduction” approach to research, now advocated by the National Institute for Health Research in the UK,^[62]^ has been shown to ensure that scientific enquiry is informed by the experiential knowledge of people on the receiving end of health research, as well as by academic wisdom.^[63]^ While the potential for coproduction approaches to shape qualitative research have been well established,^[60]^ we have shown in this study how experiential knowledge can play a role in informing the development of a trial protocol.^[43]^ In addition, it has been argued that involving the full range of stakeholders in the development of psychosocial interventions in mental health can improve the implementation and fidelity of intervention delivery,^[64]^ leading to new approaches to co-design trial interventions.^[65]^

However, given that we have noted above how the formal environment of statutory mental health services can constrain the implementation of peer support into practice,^[37,38]^ it remains to be seen if this trial will achieve fidelity in delivery of our principles-based peer support intervention. There is a lack of research testing the fidelity of peer support interventions in the context of a randomized controlled trial. One study tested the fidelity of delivery of an illness management intervention, as delivered by both peer workers and mental health professionals,^[66]^ while a tool has been developed for the informal evaluation of peer-run respite services.^[67]^ We have developed a fidelity index (in preparation) to test the fidelity of delivery of one-to-one peer support in mental health services against the domains of our *peer support principles* framework.^[44]^ We hope that this trial will lend clarity to the emerging evidence base for peer support in mental health services, offering a high-quality study of clinical and cost effectiveness of peer support for discharge from inpatient to community mental health care while specifying clearly what peer workers do and how that brings about change.

## Acknowledgments

The study sponsor is St George's, University of London, as represented by Dr Subhir Bedi, Head of Research Governance and Delivery, Joint Research and Enterprise Services, St George's, University of London, Cranmer Terrace, London, UK, sbedi@sgul.ac.uk. The study sponsor played no role in the design of the study, and will play no role in the conduct of the study, interpretation of data or decision to report the study for publication.

The study is overseen by an independent Trial Steering Committee comprising an academic chair, statistician, 1 further academic member, and 2 patient and public representatives, with responsibility for ensuring that the study is conducted to protocol and for making recommendations to the funder and sponsor for the continuation of the trial, both at end of pilot and generally; an independent Data Management Committee comprising an academic chair, statistician, and patient and public representative, with responsibility for ensuring the safe and ethical conduct of the trial and for making recommendations to the Trial Steering Committee for the continuation of the trial with respect to safety and outcome; a Lived Experience Advisory Committee comprising 8 members with personal experience of mental distress, using mental health services, and/or peer support, with responsibility for ensuring that conduct of the trial reflects the concerns of people using mental health services; a Trial Management Group comprising chief investigator, trial manager, trial statistician, health economist, patient and public representative, and other members of the study team, as appropriate, with responsibility for day-to-day operational management of the trial.

## Author contributions

**Conceptualization:** Steve Gillard, Stephen Bremner, Sarah Louise Gibson, Andrew Healey, Mike Lucock, Stefan Priebe, Julie Repper, Alan Simpson, Sarah White.

**Funding acquisition:** Steve Gillard, Stephen Bremner, Sarah Louise Gibson, Andrew Healey, Mike Lucock, Stefan Priebe, Julie Repper, Alan Simpson, Sarah White.

**Methodology:** Stephen Bremner, Sarah Louise Gibson, Stefan Priebe, Julie Repper, Alan Simpson, Sarah White.

**Project administration:** Steve Gillard, Jacqueline Marks, Stefan Priebe.

**Writing – original draft:** Steve Gillard, Stephen Bremner, Rhiannon Foster, Sarah Louise Gibson, Lucy Goldsmith, Andrew Healey, Jacqueline Marks, Rosaleen Morshead, Akshay Patel, Sarah White.

**Writing – review & editing:** Steve Gillard, Stephen Bremner, Rhiannon Foster, Sarah Louise Gibson, Lucy Goldsmith, Andrew Healey, Mike Lucock, Jacqueline Marks, Rosaleen Morshead, Akshay Patel, Stefan Priebe, Julie Repper, Sarah Roberts, Alan Simpson, Sarah White.

Steve Gillard orcid: 0000-0002-9686-2232.
